# Supplementary material for: Immunotherapy with engineered bacteria by targeting the STING pathway for anti-tumor immunity
Source: Nat Commun. 2020 Jun 1;11:2739. doi: 10.1038/s41467-020-16602-0 (PMC7264239; doi:10.1038/s41467-020-16602-0)
Supplement: Supplementary file 1 — Supplementary Information [file 41467_2020_16602_MOESM1_ESM.pdf]

Supplementary Information

Immunotherapy with engineered bacteria by targeting the STING pathway for anti-tumor immunity

Leventhal et al.

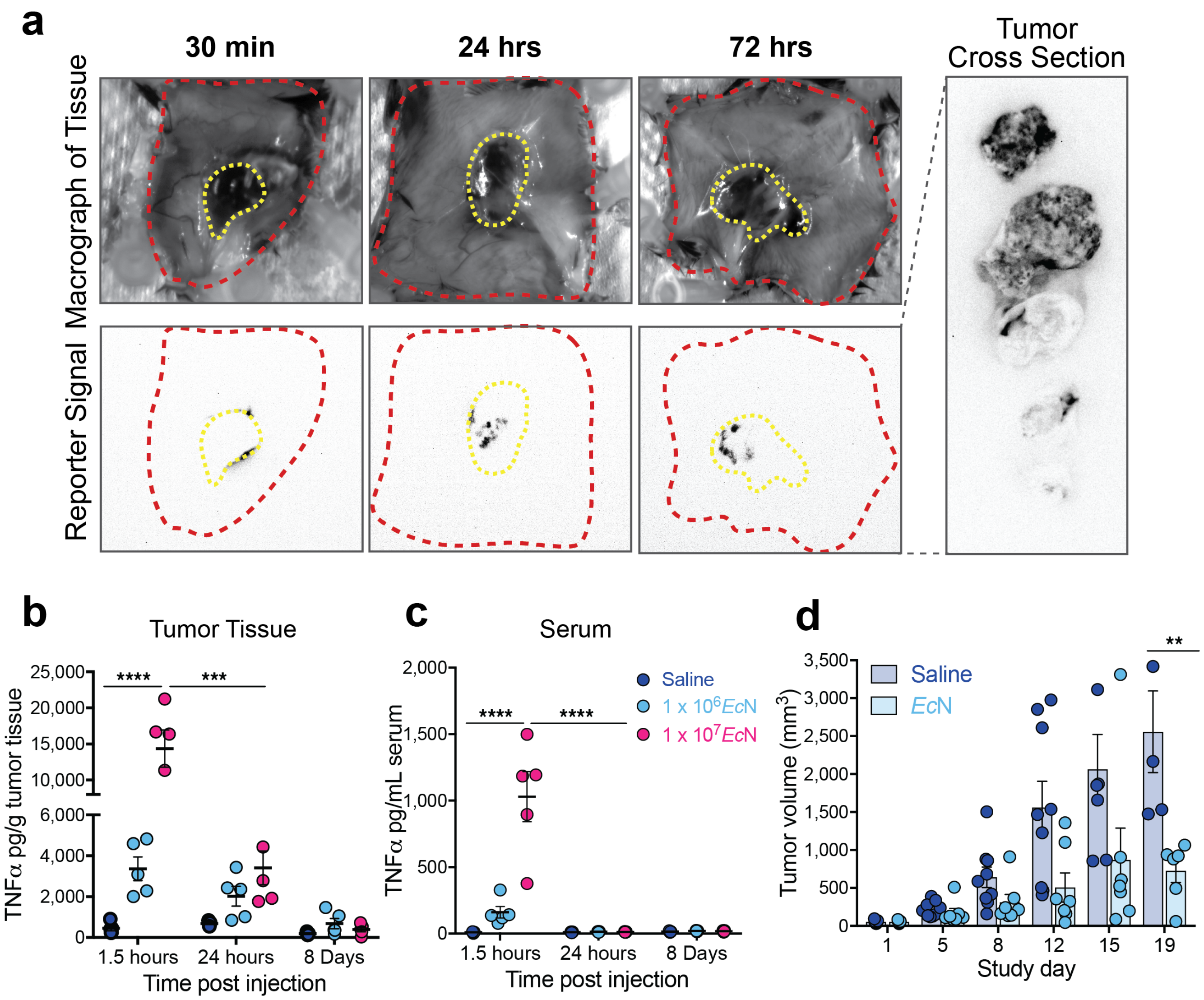

Supplementary Figure 1. ***E coli.*** **Nissle is a versatile platform for localized modulation of the tumor microenvironment. (Supplementary data for Fig. 1).** **a**, Subcutaneous B16.F10 tumors (n=3 mice per group) were established, allowed to reach 500-800 mm^3 and 1 x 10^6 CFUs of *Ec*N-LuxABCDE were intratumorally injected. At 30 mins, 24 hrs and 72 hrs post injection, tumors and surrounding healthy tissue were harvested, photographed and imaged to detect distribution of bioluminescence (reporter signal) across tumors and healthy tissue which indicates bacterial colonization. Red dotted lines represent the edge of the boarding healthy skin tissue with yellow dotted lines representing the border of the tumor. Following imaging, tumors were detached from the skin and serial sections were lined side-by-side to view bioluminescence distribution of bacteria within the tumor on macroscopic level. **b,c**, CT26 tumor-bearing mice (n=5 mice per group) were treated once i.t. as indicated. TNF-α quantification from tumor (**b**) and serum (**c**) shown at the indicated time points. (***P=0.0002, ****P<0.0001, two-way ANOVA with Tukey's multiple comparisons tests). **d**. Individual tumor volumes for Figure 1**f** (n = 9 mice for saline and n = 7 mice for *Ec*N treatment groups at the beginning of the study, **P=0.0062 for two-tailed unpaired Student's t-test comparing saline (n = 4) vs *Ec*N (n = 6) treated groups on day 19 of study). Each circle in **b-d** represents an individual animal. Data are mean with s.e.m.

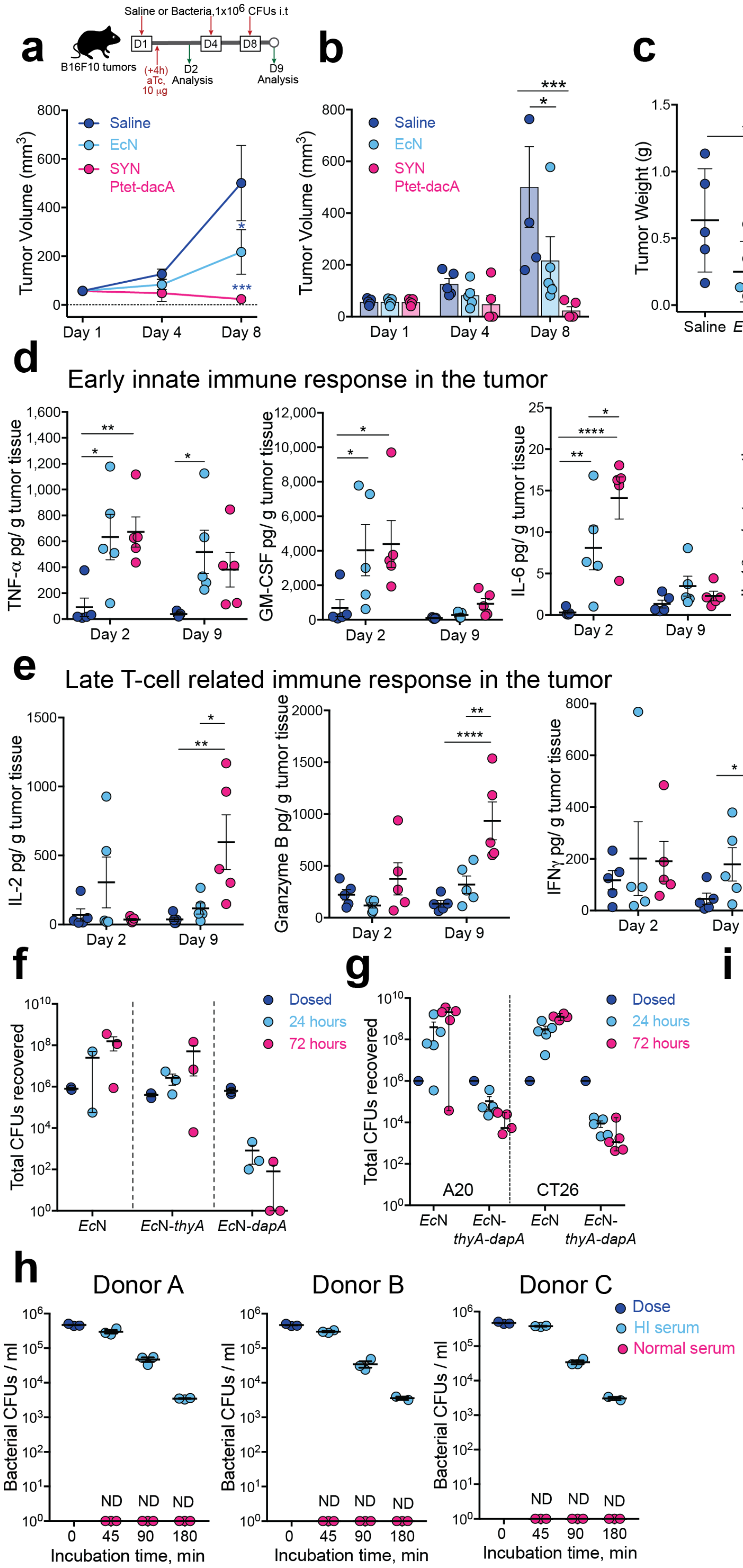

i

Antibiotic Disk	Disk Content (μg)	Zone of Inhibition Diameter (mm)	Category in accordance with CLSI* Chapter M100
		Mean +/- SD of three independent trials	
Kanamycin	30	20 +/- 1.73	Susceptible
Chloramphenicol	30	17.33 +/- 1.15	Susceptible
Ampicillin	10	15 +/- 1	Intermediate
Piperacillin-tazobactam	110	29 +/- 2.65	Susceptible
Cefepime	30	33 +/- 2.65	Susceptible
Ceftazidime	30	30 +/- 1.53	Susceptible
Aztreonam	30	33 +/- 2.08	Susceptible
Meropenem	10	36 +/- 4.36	Susceptible
Amikacin	30	24.7+/- 1.53	Susceptible
Gentamicin	120	28.7 +/- 1.53	Susceptible
Doxycycline	30	14 +/- 1	Susceptible
Tetracycline	30	18 +/- 1	Susceptible
Ciprofloxacin	5	27.3 +/- 2.08	Susceptible
Levofloxacin	5	26 +/- 1.73	Susceptible
Polymyxin B1	300	17.33 +/- 1.15	N/A
Trimethoprim-sulfamethoxazole	25	No Zone	Resistant
Negative Control	N/A	No Zone	N/A

*CLSI- Clinical and Laboratory Standards Institute

Supplementary Figure 2. Engineering a STING agonist producing live therapeuticSYNB1891 (Supplementary data for Fig. 2). a-e, B16.F10 tumor-bearing mice (n=5 per group per time point) were intratumorally (i.t.) administered with 1 x 10^6 CFUs of *Ec*N or SYN-pTet-dacA bacteria, or saline injection controls, on study days 1, 5 and 8. Four hours post i.t. injection all mice received 10 μg anhydrous tetracycline (aTc) via intraperitoneally injection

(to induce dacA expression for the SYN-pTet-dacA treated mice and as control for all other groups). Cohorts of mice were then euthanized on study day 2 and 9, with tumors harvested, weighed and homogenized to isolate tumor supernatants. Supernatants were then assayed with the panel of cytokines shown using a custom Luminex panel. **a,** Average and **b**, individual tumor volumes on days 1, 4 and 8. **c**,Tumor weight on day 9 (**a,b** *P=0.0163, ***P=0.0002, two-way ANOVA with Tukey's multiple comparisons tests; **c**, **P=0.0086, one-way ANOVA with Tukey's multiple comparisons tests). Early innate immune cytokines upregulated in tumors on day 2 are shown on panel **d** (*P= [0.015÷0.044], **P= [0.0018÷0.0089], ****P<0.0001, two-way ANOVA with Tukey's multiple comparisons tests). Late T-cell related cytokines upregulated in tumors on day 9 are shown on panel **e** (*P= [0.016÷0.038], **P= [0.0013÷0.0052], ****P<0.0001, two-way ANOVA with Tukey's multiple comparisons tests)**. f,** Total CFUs recovered from B16.F10 tumors i.t.-injected with 1×10^6 CFUs of either prototrophic *Ec*N or strains containing either a *thyA* or *dapA* auxotrophy at the indicated time-points. **g**, Total CFUs recovered from A20 or CT26 tumors i.t.-injected with 1×10^6 CFUs of either prototrophic *Ec*N or a strain containing dual *thyA* and *dapA* auxotrophies at the indicated time-points. Each circle in **b-g** represents an individual animal and data are mean with s.e.m. **h**, Sensitivity of SYNB1891 to human serum. 5×10^5 CFUs/ ml of SYNB1891 were incubated in 90% of either normal human serum (3 donors, n=3 replicates per donor per time point) or heat-inactivated (HI, at 65°C for 30 min) serum from a corresponding donor at 37°C for indicated period of time. Recovered CFUs/ ml are show for each donor. N.D. – not detected. Each circle represents an individual replicate. Data are mean with s.e.m. **i**, Antibiotic susceptibility testing of SYNB1891 for a panel of 16 antibiotics.

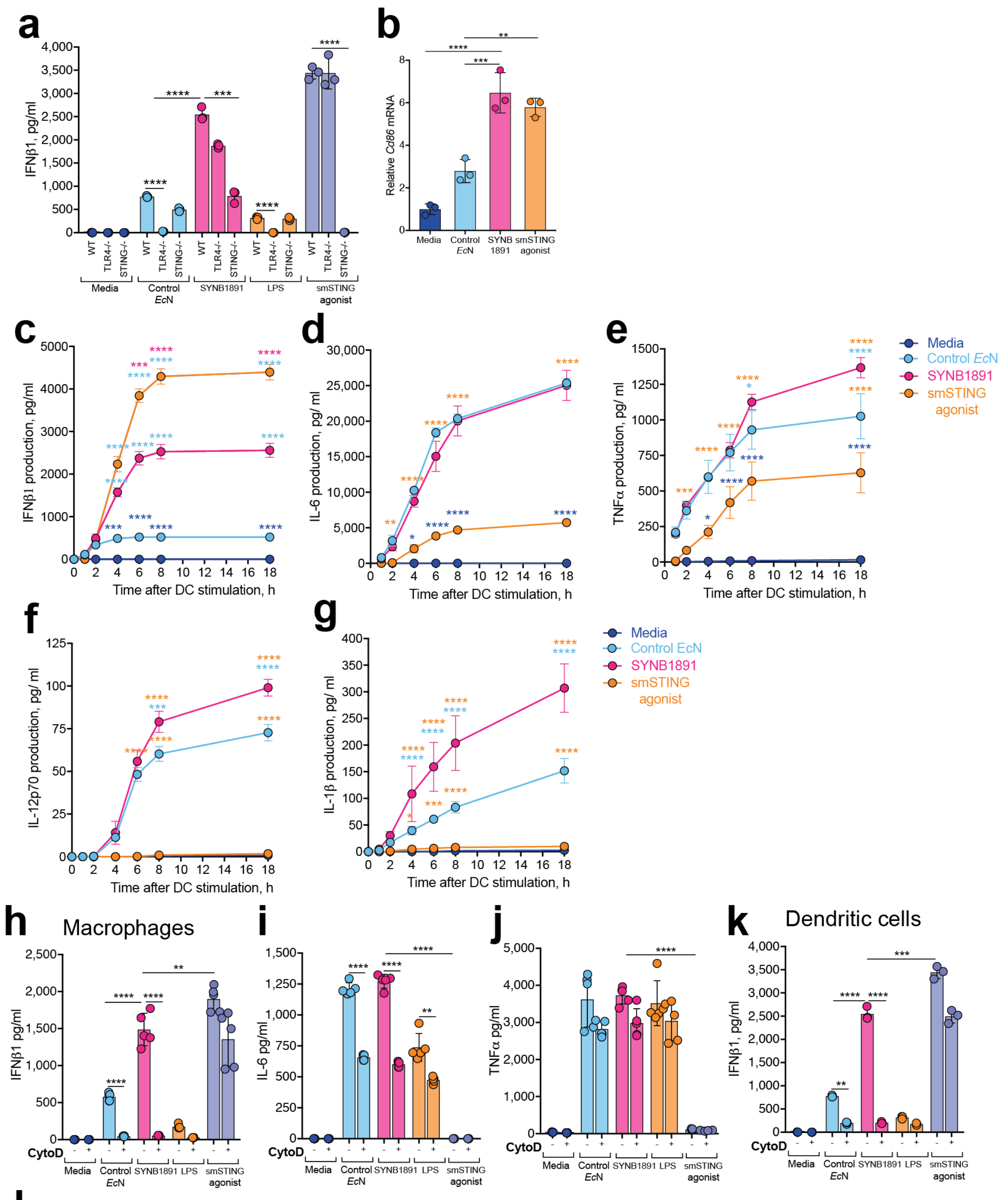

l

Innate Immune Signal	Bacterial Chassis		SYNB1891		smSTING Agonist	
	Relative Expression	Phagocytosis Dependent	Relative Expression	Phagocytosis Dependent	Relative Expression	Phagocytosis Dependent
**Type I IFN**	+	Yes	+++	Yes	++++	No
**Pro-inflammatory cytokines**						
TNFα	+++	No	++++	No	++	No
IL-6	++++	Partial	++++	Partial	+	No
IL-12p70	+	Not Determined	++	Not Determined	-	Not Determined
IL-1β	+	Not Determined	++	Not Determined	-	Not Determined
**Co-stimulation** CD86	+	Not Determined	++	Not Determined	++	Not Determined

Supplementary Figure 3. Phagocytosis- and STING-dependent induction of Type I interferon by SYNB1891 (Supplementary data for Fig. 3). a, WT, TLR4$^{-/-}$ and STING$^{-/-}$ BMDCs were treated with Control *EcN* (MOI: 25), pre-induced SYN1891 (MOI: 25), LPS (100 ng/mL), or smSTING agonist (5 μg/mL) for 4 hours (n = 3 biological replicates per group per genotype) and IFN-β1 protein secretion was quantified (***P=0.0003, ****P< 0.0001, two-way ANOVA

with Tukey's multiple comparisons tests). **b-g,** WT BMDCs were treated as described in **a** for 18 hours (n = 3 biological replicates per group). Cells were analyzed for the upregulation of CD86 mRNA (**b,** **P=0.0013, ***P=0.0003, ****P< 0.0001, one-way ANOVA with Tukey's multiple comparisons tests) and for kinetics of indicated cytokine secretion at the indicated time points (**c-g**), *P= [0.0125÷0.0223], **P=0.09, ***P= [0.0002÷0.0004], ****P<0.0001, two-way ANOVA with Tukey's multiple comparisons tests, dark blue stars – indicated group vs media, blue stars – indicated group vs control *Ec*N, pink stars – indicated group vs SYNB1891, yellow stars – indicated group vs smSTING Agonist. **h**-**j**, RAW 264.7 macrophages (n = 5 biological replicates per group) or **k**, WT BMDCs (n = 3 biological replicates per group) were treated as described in **a** for 4 hours. In the indicated groups cells were pre-treated with for 1 hour with Cytochalasin D (10μM). Macrophages or BMDCs incubated in media alone served as a negative control. Cells were analyzed for protein secretion of IFN-β1 (**h,k**), IL-6 (**i**) and TNF-α (**j**) . **h-k**, **P= [0.001÷0.008], ***P= 0.0002, ****P<0.0001, two-way ANOVA with Tukey's multiple comparisons tests. **(a-k**) Data are representative of two or more independent experiments per cell type with mean and s.d. shown. Each circle represents an independent experimental replicate. **l**, Table summarizing the data shown in Fig. 3 and Supplementary Fig. 3. This table illustrates the relative expression levels of Type I IFN (IFN-β1), other proinflammatory cytokines and co-stimulatory markers (CD86) following treatment with SYNB1891, non-engineered *Ec*N or smSTING-agonist.

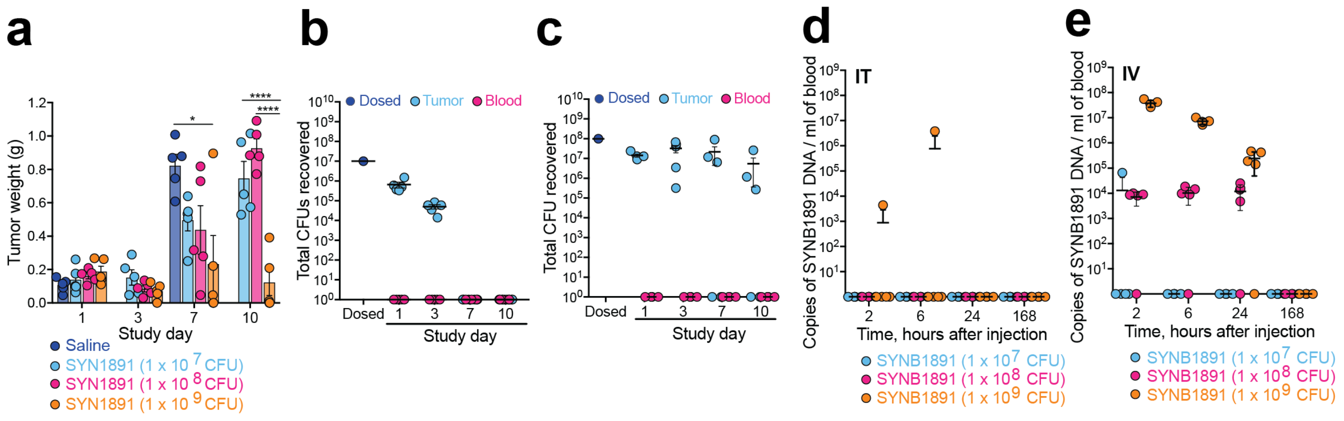

Supplementary Figure 4. ***In Vivo* dynamics of SYNB1891 in tumor bearing mice (Supplementary data for Fig. 4).** B16.F10 tumor-bearing mice (n=5 mice per bacterial group per time point, n=5 per saline group for day1 and day 7) were treated with a single i.t. dose of either saline, 1 x 10^7 or 1 x 10^8 CFUs SYNB1891 on Study Day 0. (**a**) Individual tumor volumes at different time points (* P=0.0163, one-way ANOVA with Tukey's multiple comparisons tests for Day 7 ; **** P < 0.0001, two-way ANOVA for bacterial groups with Tukey's multiple comparisons tests. Bacterial abundance within tumor homogenates and blood at the indicated timepoints post-i.t. injection with 1 x 10^7 (**b**) and 1 x 10^8 (**c**) doses. SYNB1891 was either intratumorally (IT, **d**) injected into B16.F10 tumor bearing mice or as a positive control for bacterial abundance in blood, intravenously (IV, **e**) injected into healthy age-matched controls at the indicated doses (n=5 mice per group per time point). Blood was then harvested at the indicated time points and the presence of SYNB1891 bacterial DNA was detected by SYNB1891 probe specific qPCR. (**a-e**) Data are representative of two or more independent experiments with mean and s.e.m. shown. Each circle represents an individual animal.

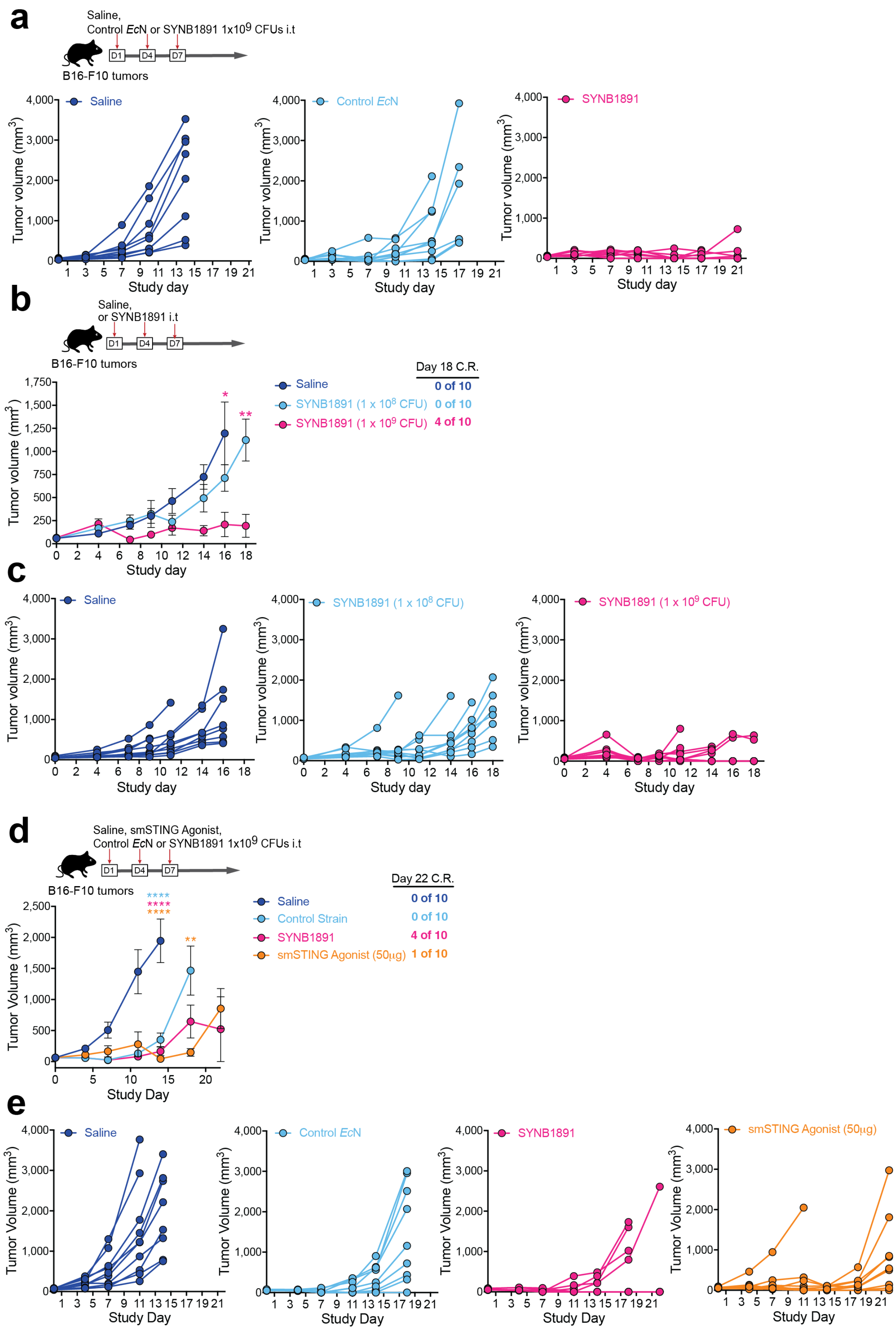

Supplementary Figure 5. SYNB1891 treatment triggers efficacious antitumor immunity and immunological memory (Supplementary data for Fig. 5). a. On Study days 1, 4 and 7, B16.F10 tumor-bearing mice (n=10 mice per

group) were treated with i.t. doses of either saline (injection control), 1 x 10^9 CFUs SYNB1891 or 1 x 10^9 CFUs of Control *Ec*N lacking the P_{fnrs}-*dacA* circuit. Individual tumor growth data is shown. **b,c** On Study days 1, 4 and 7 B16.F10 tumor-bearing mice (n=10 mice per group) were treated with i.t. doses of either saline (injection control), 1 x 10^8 CFUs SYNB1891 or 1 x 10^9 CFUs of SYNB1891. Representative tumor growth data is shown with the ratio of complete responders (C.R.) or mice having no palpable tumor on Study day 18 (**b**, * P=0.03 (pink stars - indicated group vs 1 x 10^9 CFUs of SYNB1891) one-way ANOVA with Tukey's multiple comparisons tests for Day 16; ** P =0.0059, two-tailed unpaired Student's *t*-test of two SYNB1891 treatment groups at Day 18), or as individual tumor growth (**c**). (**d, e**) On Study days 1, 4 and 7 B16.F10 tumor-bearing mice (n=10 mice per group) were treated with i.t. doses of saline (injection control), 1 x 10^9 CFUs of Control *Ec*N lacking the P_{fnrs}-*dacA* circuit, 1 x 10^9 CFUs SYNB1891 or 50μg small molecule STING agonist (smSTING Agonist). Doses were separated by three days. Representative tumor growth data is shown with the ratio of C.R. on Study day 22. Average tumor volumes are shown for each group up until the date where greater than 50% of mice are taken off study (**d**, **** P<0.0001 (blue stars – indicated group vs control *Ec*N, pink stars – indicated group vs SYNB1891, yellow stars – indicated group vs smSTING Agonist) one-way ANOVA with Tukey's multiple comparisons tests for Day 14; ** P =0.0066 (yellow stars – indicated group vs smSTING Agonist) one-way ANOVA with Tukey's multiple comparisons tests for Day 18). Individual tumor growth data is shown (**e**). (**a-e**) Data are representative of two or more independent experiments with mean and s.e.m. shown (**b,d**) or each circle as an individual animal.

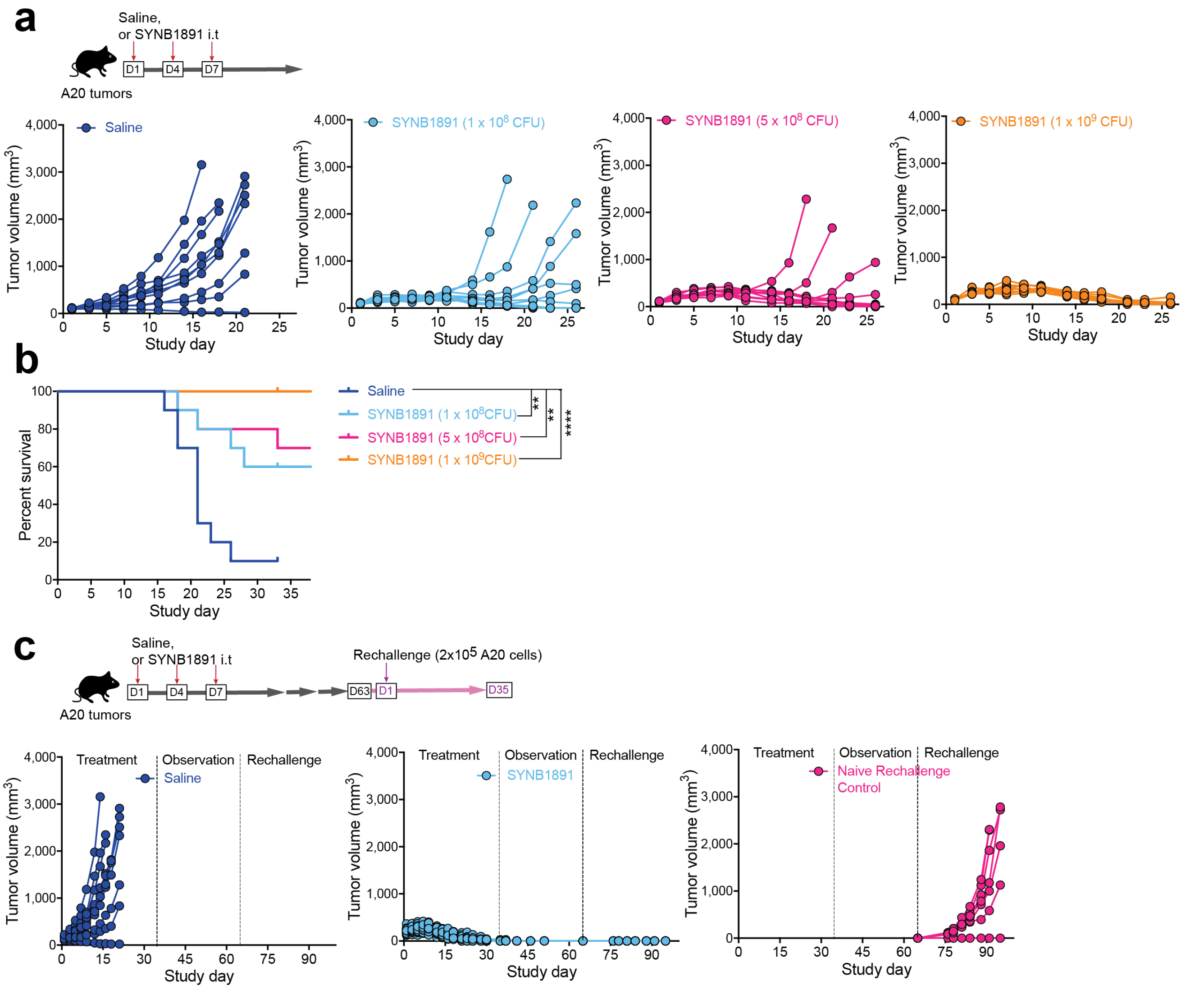

Supplementary Figure 6. SYNB1891 treatment triggers efficacious antitumor immunity and immunological memory (Supplementary data for Fig. 5). a,b, On Study days 1, 4 and 7, A20 tumor-bearing mice (n=10 mice per group) were treated with i.t. doses of either saline or varying quantities of SYNB1891as indicated. Individual tumor growth data (**a**) and long-term survival (**b,** **P=[0.0043÷0.0084], ****P<0.0001, Mantel-Cox log-rank comparisons for the indicated groups) are shown. Data are representative of three independent experiments. **c**, Mice (n = 10 per group) treated as described in **a** from all SYNB1891 treatment groups which remained tumor free by Study day 50 were rechallenged by subcutaneous injection of A20 cells on the contralateral flank alongside naïve age-matched controls. Individual tumor volumes are presented. (**a-c**) Data are representative of two or more independent experiments, each circle represents an individual animal.

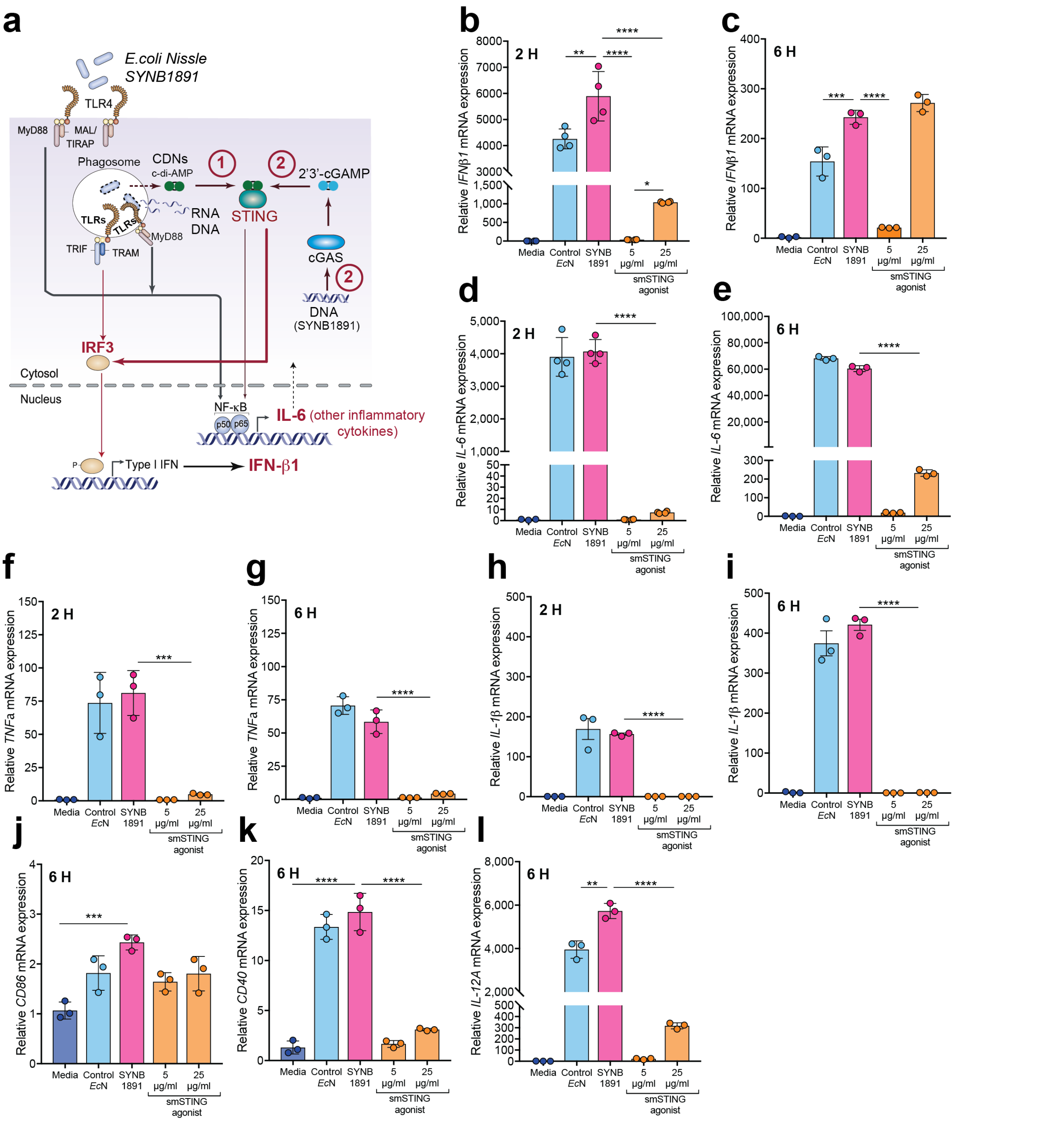

Supplementary Figure 7. SYNB1891 Activity in Human Antigen-presenting Cells (Supplementary data for Fig. 6). a, Illustration for the proposed mechanism of action for SYNB1891 in human antigen presenting cells (APCs). STING activation in human APCs by SYNB1891 consists of 2 signals: (1) direct STING activation by bacterial c-di-AMP and (2) indirect activation through bacterial DNA- cGAS-STING axis. Other bacterial ligands (such as LPS) readily available in gram- negative *E.coli Nissle* stimulate proinflammatory signaling through a variety of pattern recognition receptors and NF-κB activation. **b-l,** Monocyte-derived primary human DCs were treated with Control *Ec*N (MOI: 25), pre-induced SYN1891 (MOI: 25), LPS or smSTING agonist (5 and 25 μg/mL) for 2 and 6 hours (n=3 replicates per group per time point). Human DCs incubated in media alone served as negative control. Cells were analyzed for the early upregulation of *IFNB1* (**b**), *IL6* (**d**), *TNFα* (**f**) or *IL1β* (**h**) mRNA at 2 hours (2 H) and late upregulation of *IFNB1* (**c**), *IL6* (**e**), *TNFα* (**g**), *IL1β* (**i**), *CD86* (**j**), *CD40* (**k**) and *IL-12A* (**l**) mRNA at 6 hours (6 H). Data are mean with s.d. Each circle represents an independent experimental replicate. *P= 0.0474, **P= [0.0011÷0.0044], ***P= [0.0002÷0.0005],****P<0.0001, one-way ANOVA with Tukey's multiple comparisons tests.

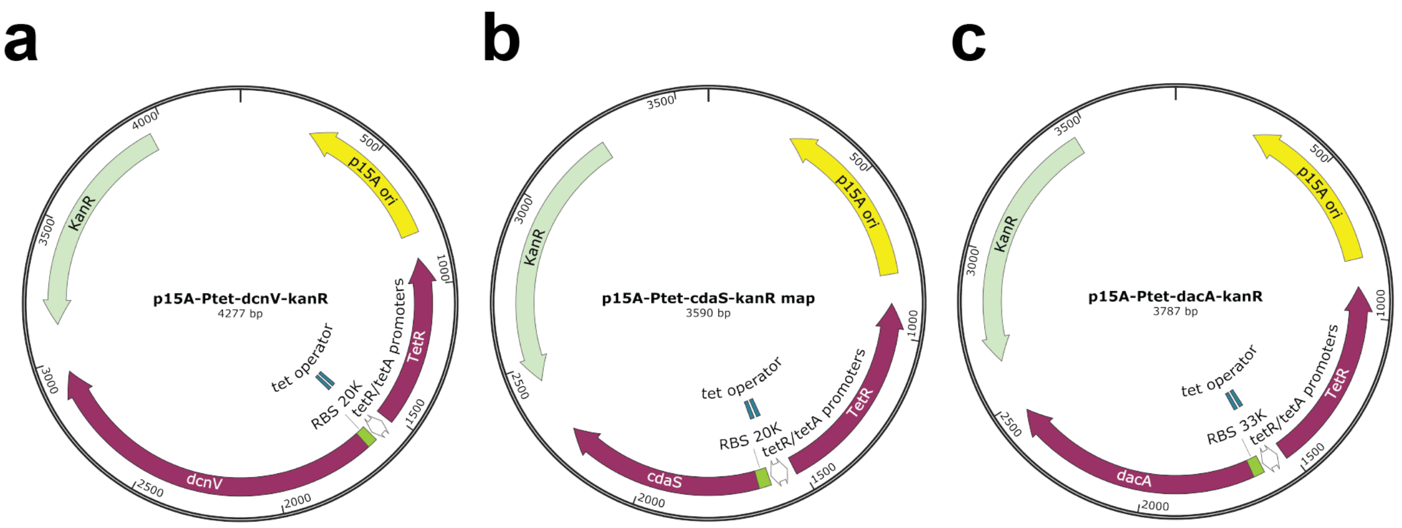

Supplementary Figure 8. Maps of STING agonist (CDA)-producing plasmids used in this study. a, p15A plasmid with expression of cyclic dinucleotide synthetase DcnV under the control of Ptet promoter. **b,** p15A plasmid with expression of cyclic dinucleotide synthetase CdaS under the control of Ptet promoter. **c,** p15A plasmid with expression of cyclic dinucleotide synthetase DacA under the control of Ptet promoter.

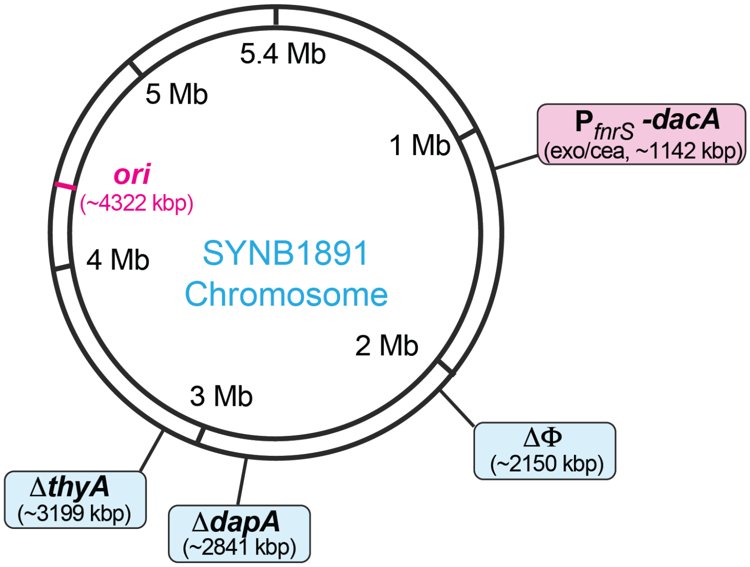

Supplementary Figure 9. Map of SYNB1891 Genome. The locations of the genomic modification sites in SYNB1891 are shown, with kbp designation indicating the chromosomal position relative to the 0/5.4 Mb reference marker. The chromosomal origin of replication is shown as a pink line. Pink text box designates *dacA* gene insertions and light blue text boxes with *Δ* symbol designate the locations of the *dapA*, *thyA* and *Φ* deletion. Gene names in parenthesis refer to the upstream and downstream genes surrounding the inserted genes.

Abbreviations: *exo/cea* = intergenic region between the *exo* and *cea* genes; kbp = kilo base pair; *dacA* = cyclic-di-AMP synthase from *Listeria monocytogenes*; P_{fnrS} = anaerobic inducible promoter activated by the fumarate and nitrate reductase transcriptional activator, FNR; *ΔdapA* = deletion of *dapA* gene (leading to auxotrophy); *ΔthyA* = deletion of *thyA* gene (leading to auxotrophy); *ΔΦ* = partial deletion of endogenous Nissle prophage

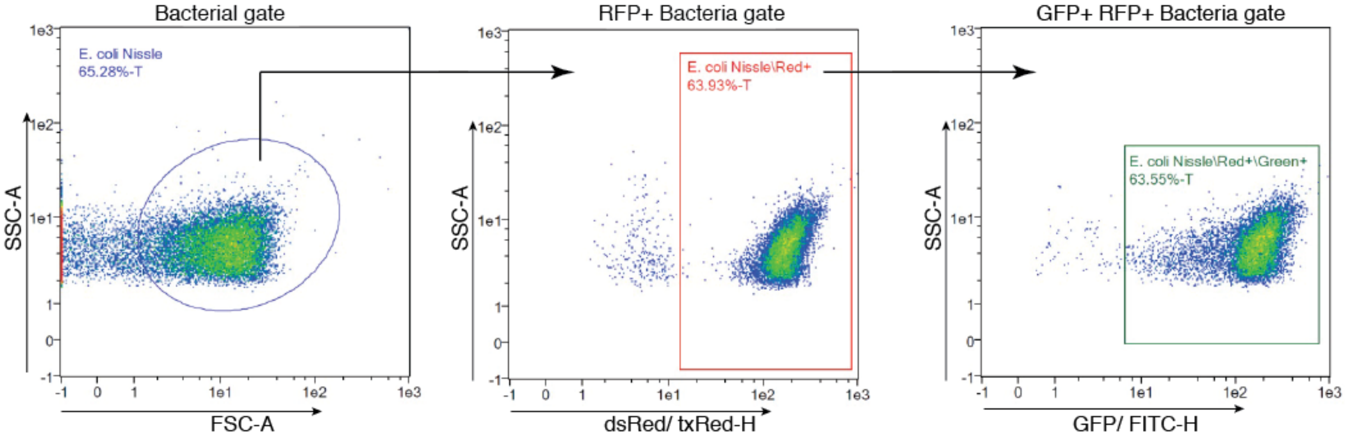

Supplementary Figure 10. Flow cytometry gating strategy for bacteria, example. This example corresponds to data in Fig.2**d**. Macsquant VYB flow cytometer was used to analyze the samples and collected the data. The data were analyzed using MACSQuantify software provided by Miltenyi Biotec. Detector settings: FSC = 580V, SSC = 340V, Y2 (red) = 820V, B1 (green) = 660V. The FSC and SSC were set up specifically for the visualization of E. coli Nissle. The gating strategy was set up with E. coli Nissle strains with no fluorescent proteins (negative control), with red fluorescent protein only (single positive control), or with red and green fluorescent proteins expressed in one strain (double positive control).

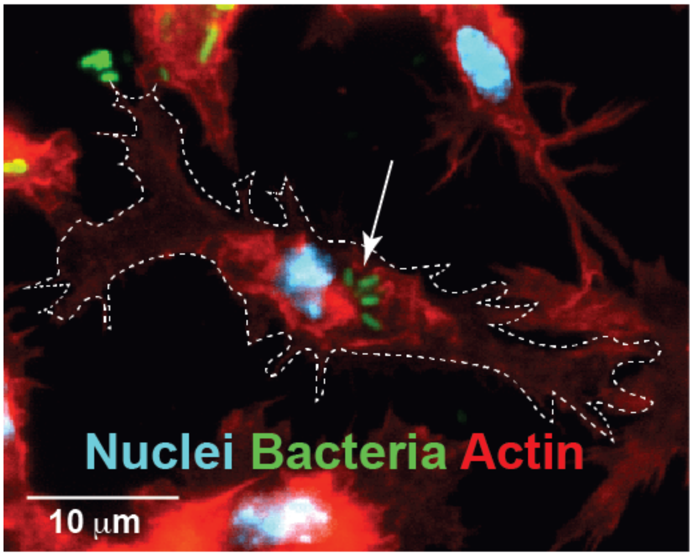

Supplementary Figure 11. Phagocytosed bacteria quantification example. This example corresponds to microscopy data in Fig.3 d-g and is representative of an individual DC containing phagocytosed bacteria from 7 FOV from 2 independent experiments. For phagocytosis analysis, WT BMDCs were incubated with pre-induced SYNB1891-*gfp* (MOI: 25) for 1h in control media and non-internalized bacteria were washed out. Cells were stained for microscopy: cell nuclei were labeled with Hoechst (Blue), F-actin was stained with ActinRed 555 probe (Red), SYNB1891-*gfp* were labeled with anti-GFP (Green). The outline of dendritic cell labeled with ActinRed 555 is shown in white dotted line. White arrow points to the bacteria (in green) localized within the cellular border and surrounded by F-actin. These bacteria were counted as internalized by dendritic cell.

Supplementary Table 1. List of strains used in this publication

Name/Reference	Synlogic Strain #	Genotype	Description	Activity Induction
Not Referenced	SYN001	Control bacterium	N/A	N/A
*Ec*N	SYN094	SYN001 with strep resistance	Control bacterium with strep resistance	N/A
*Ec*N-*LuxABCDE*	SYN5353	SYN94, pST-constitutive-*luxABCDE-cmR*	Bacterium with constitutively expressed luminescence producing pathway enzymes LuxA, LuxB, LuxC, LuxD and LuxE	N/A
SYN-P_{tet}-*dncV*	SYN3526	SYN001, p15A-P_{tet}-*dcnV-kanR*	Bacterium with plasmid containing cyclic dinucleotide synthetase DcnV	aTc
SYN-P_{tet}-*dacA*	SYN3527	SYN001, p15A-P_{tet}-*dacA-kanR*	Bacterium with plasmid containing cyclic dinucleotide synthetase DacA	aTc
SYN-P_{tet}-*cdaS*	SYN3528	SYN001, p15A-P_{tet}-*cdaS-kanR*	Bacterium with plasmid containing cyclic dinucleotide synthetase CdaS	aTc
SYN-*mcherry*-P_{tet}-*gfp*, P_{tet}-*gfp*	SYN4360	SYN001, *malE/K*::$c_{constitutive}$-*mCherry-cmR*, p15A-P_{tet}-*gfp-kanR*	Bacterium with constitutively expressed mCherry and plasmid containing gene encoding GFP under the control of Ptet promoter	aTc
SYN-*mcherry*-P_{sal}-*gfp*, P_{sal}-*gfp*	SYN4352	SYN001, *malE/K*::$P_{constitutive}$-*mCherry-cmR*, p15A-P_{sal}-*gfp-kanR*	Bacterium with constitutively expressed mCherry and plasmid containing gene encoding GFP under the control of Psal promoter	salicylate
SYN-*mcherry*-P_{cmt}-*gfp*, P_{cmt}-*gfp*	SYN4353	SYN001, *malE/K*::$P_{constitutive}$-*mCherry-cmR*, p15A-P_{cmt}-*gfp-kanR*	Bacterium with constitutively expressed mCherry and plasmid containing gene encoding GFP under the control of Pcmt promoter	cumate
SYN-*mcherry*-P_{fnrS}-*gfp*, P_{fnrS}-*gfp*	SYN4359	SYN001, *malE/K*::$P_{constitutive}$-*mCherry-cmR*, pSC101-P_{fnrS}-*gfp*-$P_{constitutive}$-*mcherry-ampR*	Bacterium with constitutively expressed mCherry and plasmid containing gene encoding GFP under the control of PfnrS promoter	anaerobic
SYN-P_{fnrS}-*dacA*	SYN4448	SYN094, p15A-P_{fnrS}-*dacA-kanR*	Bacterium with plasmid containing gene encoding cyclic dinucleotide synthetase DacA under the control of PfrnS promoter	anaerobic
*Ec*N-*thyA*	SYN1534	SYN001, Δ*thyA*-cmR	Control bacterium with thymidine auxotrophy	N/A
*Ec*N-*dapA*	SYN766	SYN001, Δ*dapA*-cmR	Control bacterium with diaminopimelic acid auxotrophy	N/A
*Ec*N-*thyA-dapA*, Control *Ec*N	SYN4740	SYN001, Δ*thyA*, Δ*dapA*, Δ*Φ*	Control bacterium with phage fragment knockout and thymidine and diaminopimelic acid auxotrophy	N/A
SYNB1891-cmR	SYN4910	SYN001, Δ*thyA-cmR*, Δ*dapA*, Δ*Φ*, *exo/cea*::-P_{fnrS}-*dacA*	Bacterium with integrated *dacA* gene under the control of PfnrS promoter, phage fragment knockout and thymidine and diaminopimelic acid auxotrophy	anaerobic
SYNB1891-*gfp*	SYN5674	SYN001, Δ*thyA-cmR*, Δ*dapA*, Δ*Φ*, *exo/cea*::-P_{fnrS}-*dacA*, pSC101-P_{cI}-*gfp-ampR*	Bacterium with integrated *dacA* gene under the control of PfnrS promoter, plasmid containing gene encoding GFP under the control of constitutive promoter, phage fragment knockout and thymidine and diaminopimelic acid auxotrophy	anaerobic
SYNB1891	SYN4933	SYN001, Δ*thyA*, Δ*dapA*, Δ*Φ*, *exo/cea*::-P_{fnrS}-*dacA*	Bacterium with integrated *dacA* gene under the control of PfnrS promoter, phage fragment knockout and thymidine and diaminopimelic acid auxotrophy without any antibiotic marker	anaerobic

Supplementary Table 2. DNA sequences for STING agonist (CDA)- producing enzymes

Name/Reference	DNA sequence
dcnV	ATGCGCATGACGTGGAACTTCCATCAATATTATACAAACCGCAACGATGGCTTAATGGGTAAATT GGTTTTGACCGATGAAGAAAAGAATAATTTGAAAGCTTTGCGTAAGATTATCCGCCTTCGCACGC GCGACGTGTTTGAGGAAGCAAAGGGCATCGCCAAAGCCGTAAAGAAAAGCGCGTTGACTTTTGA GATCATCCAGGAGAAGGTTAGCACGACGCAAATTAAACATCTTTCCGATAGTGAGCAGCGCGAG GTAGCTAAGTTAATCTACGAAATGGATGATGACGCGCGTGATGAATTCCTGGGTCTTACTCCCCG TTTCTGGACACAAGGCTCGTTCCAATACGACACCCTTAATCGTCCATTTCAACCAGGACAGGAAA TGGACATTGACGACGGAACGTATATGCCGATGCCCATTTTTGAGAGTGAACCCAAGATTGGTCAT TCGCTTTTGATTTTACTGGTAGACGCCAGCTTAAAAAGCTTAGTCGCTGAAAATCATGGCTGGAA ATTCGAAGCAAAGCAAACTTGCGGGCGTATTAAGATCGAAGCAGAAAAAACCCATATCGACGTA CCTATGTACGCGATTCCGAAGGATGAATTCCAGAAAAAGCAAATCGCTCTTGAAGCCAACCGCT CGTTTGTGAAAGGGGCGATCTTTGAATCATACGTTGCAGACAGTATCACTGATGACTCCGAGACG TATGAGTTGGACTCTGAGAATGTTAATTTAGCTTTACGCGAAGGTGATCGCAAATGGATTAATTC AGACCCCAAAATTGTAGAAGATTGGTTCAACGACTCCTGTATCCGCATTGGTAAGCACCTGCGTA AAGTTTGCCGTTTTATGAAGGCTTGGCGTGACGCCCAGTGGGACGTTGGAGGGCCTTCGTCTATC TCTTTGATGGCCGCGACTGTGAATATTTTAGACTCTGTTGCACACGATGCAAGCGACCTGGGCGA AACCATGAAGATTATCGCTAAGCATTTACCCTCCGAGTTCGCACGTGGGGTAGAATCCCCCGACT CGACGGATGAGAAACCTCTGTTTCCTCCATCTTATAAACACGGTCCACGTGAGATGGACATTATG AGCAAGCTTGAGCGCCTTCCAGAAATCCTGTCAAGTGCTGAATCGGCTGACAGTAAGTCTGAGG CCCTGAAAAAAATTAACATGGCGTTTGGGAATCGTGTGACCAATTCTGAACTGATTGTCCTGGCT AAAGCTCTTCCAGCCTTTGCTCAGGAGCCTTCAAGCGCTAGTAAGCCAGAAAAGATTTCGTCAAC GATGGTGTCTGGGTGA
cdaS	ATGAAAGCTATGCGCTACGAACAAATTTCCGAAAACGCTTTCAAAGGGAAGATTCAAGTTTATCT GGAGCAAATTTTGGGTGATGCTTCCCTTATCTTAAAGACACTGCACGAGAAAGACCAGTGTTTAC TTTGTGAGCTGGACGACCTTGGACATGTCTTTCAGGACATGCAAGGTATCGCGTCCTCCTTTTATT TACAAAGCTACATTGAGGAATTTACACCGGCGTTCATCGAACTGGCCAAGGCAATCAAAGCTCT GAGCGAACATAAGCACGGTGCCTTGATTGTGATTGAGCGCGCGGACCCGGTCGAGCGTTTCATC CAGAAAGGCACGTCATTACATGCAGAAATCTCCAGCTCGCTTATCGAGTCAATTTTCTTTCCGGG CAACCCACTGCATGATGGTGCCCTTCTGGTCCGCGAAAATAAGTTAGTGTCAGCGGCTAACGTAC TGCCGCTTACAACCAAAGAGGTGGACATTCACTTGGGCACGCGCCATCGCGCGGCGTTAGGTAT GTCAGGGTACACCGACGCCCTTGTCTTAGTGGTGTCGGAAGAAACGGGTAAGATGAGTTTCGCC AAAGATGGAGTCTTGTACCCTTTAATCTCCCCACGCACCTGA
dacA	ATGGACTTTTCCAACATGAGTATCCTTCACTATTTAGCGAATATTGTAGACATTTTGGTGGTTTGG TTCGTAATTTACAAGGTTATCATGCTTATCCGCGGCACGAAAGCCGTCCAGCTGTTGAAGGGGAT TTTCATTATTATTGCCGTCAAGTTACTTAGCGGCTTCTTCGGATTGCAGACAGTGGAATGGATTAC TGATCAAATGCTGACTTGGGGGTTTCTTAGCCATTATCATTATCTTTCAACCGGAATTGCGTCGTGC CCTGGAGACTTTGGGGCGTGGCAATATCTTTACCCGCTATGGATCACGCATTGAGCGTGAACAAC ACCACCTTATTGAGTCTATTGAAAAGTCCACGCAATACATGGCGAAGCGTCGCATTGGAGCTTTG ATCTCTGTGGCTCGTGATACAGGCATGGACGACTACATCGAGACTGGCATTCCGCTTAATGCGAA AATCTCATCGCAATTATTGATTAATATCTTCATCCCCAATACCCCTCTTCACGATGGCGCAGTGAT CATTAAAGGTAACGAGATCGCGAGCGCTGCCAGTTATCTGCCATTGTCCGACTCGCCGTTTCTTT CTAAGGAGCTGGGAACTCGCCATCGTGCCGCATTAGGGATTTCCGAGGTGACAGATTCAATCAC AATCGTTGTCAGCGAGGAAACAGGTGGGATTTCCCTTACGAAGGGAGGCGAACTGTTCCGTGAT GTATCCGAAGAAGAATTGCATAAGATTTTGCTTAAAGAGCTGGTGACTGTCACAGCTAAAAAAC CAAGCATTTTCTCCAAATGGAAAGGAGGTAAGTCCGAGTGA

Supplementary Table 3. Sequence Analysis of the *exo/cea*::P_{fnrS}-*dacA* insertion

DNA Sequence	Comments
gcccttataactcgaaatacagccagcaatacgctgatgcgtatggccttctgccagcgctcgtttttccagagc<br>gtgccattcagctccaactcgtgtcaggcgtagtttacgcaggctgcgttcaaggtgatggatattgctcactgc<br>gaacctcccttgccacgcagtagttgctcataactgctcaggttatgctgtatcacattgactactggcaacgcct<br>tttcctttatacccaggaagcgcatcagccggtgcagatccacgtttcccggggggattcaacatctgctccatac<br>cttttgccacgactgagatatcgtcgtatcctgcagccagtttcagggcatgtaccatcagccttcctgccatgtc<br>ggactccagatgattacataagcgacgccacagcctccgcaattcatcgtctggcaggatgtcatttctcagcg<br>ttgcatggcagaacgcaccgggctttttgccagactgtcgatcacatgtcggaagtcgatacgacgggcccg<br>cgtcttccctttttctggtctgacacgagggcagctcatgacctcgctgctgccaacgtaacggctcagacgatc<br>gtcccataaccggacccgtaacagttgacctacaagccgggaaggtacgctgtagacgatgtgcttcacattg<br>atggtactgctgcggctaaccctcacagtcagcactgcgacgaagcggcagcggtttcagatgaagacgttct<br>tccttgatcagatcatgattgttacggttgtgccgcataacctgctgagtgatgaaggcctgatattcttctatggt<br>gctgaagtcgttactgccccgcagtatcagcgcctgacagatacgccttttcagatgcccgtgggcactttcaa<br>ccgagccattttcgtggccccgaccggaattattatgtacgccctgcattccgtagtgctgacagatagcagtat<br>aacgctcagtcagctcgcggcgtccatct**CCGCCCAGTTGTCCGAGGGCTTCCTGCAGAC**<br>**CTTCAGCCAGAGCAGAGAAGCTCTTACCACCCAGCACAACCCGCATCCAGC**<br>**TCCAGTGGCTCCATTCCAGACGGAAGTGATACAACTTACCGACGATGGTGA**<br>**CAACTACACCTTTCAGTTCAGTAAAGTCCGACAGGCCTCGCAGACCGGGCT**<br>**GATGTCGCTGGCGGAACATGACCTCCTGCTCTGCACTATACTGTAGCTTCCA**<br>**TTCGCGAACCCGCCGTTGCATTGTTCTTCGAAGGCTGTTGGGATACTGGCC**<br>**GGGATATTTATCCTGTAGCATCTCCAGCAGAGTTGTTGGTGTCAGAGCCGG**<br>**CCTCTCTTTCAACAGAGGAACAAGCATGCTGTCCCACATAGCTTCCAGAGG**<br>**AAGCTTTGCGTGTGCGCCAGTGCCGAACACTGTTGTTTTCCCACTCTCCTTT**<br>**TTCGATCCGACGACCAGAATGGACTGAGATACCAGCCTTCATGGCCGAGAT**<br>**ATGCGGTCGGTCAGGTGGTATTTGAAACCAGACTGAAGCCCACGGTCGTCA**<br>**TCGGGACGCAGGCCGCCGCCGTGCATGGTATCAGTGAGCAGGCACTCTGCG**<br>**GAGCACCATCATGGACTGATGTGGTGCGGCAACTGCGTCATGCAATCGGGG**<br>**ACCGACCAGTAATTATCTTTAATGCCCGGTTCGACATCCGCATTCTGAAAAA**<br>**GACTGCTGCCGCACATAGCGATCCGGCTGACTGGCTGGAAGAACTGACGGT**<br>**ATATTGTGTGATGGAGCTGGCTGCAGGATATTATGGAGCCTCCAACCGCTA**<br>**TGGCACTATTTCACTGGCCTGTGCTGCCAGCCAGACCGGACTGAACTGGGA**<br>**AGGGCAGGCACACTCAGCGATCGCTGACGCACGGATGACGGCAGGGGTGG**<br>**TAAACGCTATTGCTGCATATCATCTGGAACTGCTGCAGGAACAGGCACGGC**<br>**TGAAAACCTGACTGCCTGGCCTGTATACCGCAATCATTTACGTTATCCAGCG**<br>**ATTGTGTAGGCTGGGTGAATAGCTGATAAAGCGTTCGCGCTGCATTCGGCA**<br>**GTTTAATTAAAGTTGTTCTTATTGGTGGTGTTGCTTTATGGTTGCATCGTAG**<br>**TAAATGGTTGTAACAAAAGCAATTTTTCCGGCTGTCTGTATACAAAAACGCC**<br>**GCAAAGTTTGAGCGAAGTCAATAAACTCTCTACCCATTCAGGGCAATATCTC**<br>**TCTTGGATCCAAAGTGAACCCGCATCCCACGCTAACTAATATAAGGGGGGC**<br>**AAGAATGGACTTTTCCAACATGAGTATCCTTCACTATTTAGCGAATATTGTA**<br>**GACATTTTGGTGGTTTGGTTCGTAATTTACAAGGTTATCATGCTTATCCGCG**<br>**GCACGAAAGCCGTCCAGCTGTTGAAGGGGATTTTCATTATTATTGCCGTCAA**<br>**GTTACTTAGCGGCTTCTTCGGATTGCAGACAGTGGAATGGATTACTGATCAA**<br>**ATGCTGACTTGGGGTTTCTTAGCCATTATCATTATCTTTCAACCGGAATTGC**<br>**GTCGTGCCCTGGAGACTTTGGGGCGTGGCAATATCTTTACCCGCTATGGAT**<br>**CACGCATTGAGCGTGAACAACACCACCTTATTGAGTCTATTGAAAAGTCCAC**<br>**GCAATACATGGCGAAGCGTCGCATTGGAGCTTTGATCTCTGTGGCTCGTGA**<br>**TACAGGCATGGACGACTACATCGAGACTGGCATTCCGCTTAATGCGAAAAT**<br>**CTCATCGCAATTATTGATTAATATCTTCATCCCCAATACCCCTCTTCACGAT**<br>**GGCGCAGTGATCATTAAAGGTAACGAGATCGCGAGCGCTGCCAGTTATCTG**<br>**CCATTGTCCGACTCGCCGTTTCTTTCTAAGGAGCTGGGAACTCGCCATCGTG**<br>**CCGCATTAGGGATTTCCGAGGTGACAGATTCAATCACAATCGTTGTCAGCG**<br>**AGGAAACAGGTGGGATTTCCCTTACGAAGGGAGGCGAACTGTTCCGTGATG**<br>**TATCCGAAGAAGAATTGCATAAGATTTTGCTTAAAGAGCTGGTGACTGTCAC**<br>**AGCTAAAAAACCAAGCATTTTCTCCAAATGGAAAGGAGGTAAGTCCGAGTG**	The uppercase, bolded blue letters represent the engineered region of the EcN genome, including the *dacA* insertion and the EcN sequence homology arms used for homologous recombination into the EcN genome<br><br>Lowercase black letters include EcN sequence upstream and downstream from the intended genomic insertion location<br><br>The entire listed sequence here is confirmed by WGS

**AGGAGGAACGATTGGTAAACCCGGTGAACGCATGAGAAAGCCCCCGGAAG<br>ATCACCTTCCGGGGGCTTTTTATTGCGCGGACCAAAACGAAAAAAGACGC<br>TCGGGAGGAGCTGCTTCGAAGTTCCTATACTTTCTAGAGAATAGGAACTTC<br>GGAATAGGAACTAAGGAGGGACGTAAATATATCAATAAATAGCAATCCCCC<br>AGATACCTAATGTAGTTCCAGCAAGCACGGCCGGGCAGCTTGTTCTGCCTG<br>CGTTTTCTTCAATTGAGCAGTAGACCATTTAGCTGTGGCATGAATGGCTGCA<br>GAACTTTCACTGTTGCTACCTCCAGTTCCACCACCGCTGCCAGAGCCACTCC<br>CGTCTGGATTATCATTCAAAAGAGTAATGATTACCTGCCCCTTATCATCATA<br>AGGAACACCATCTTTATAGTACGCTACAGCTGTTTCCATTATAAAATCCTCT<br>TTGACATTAAAAACAATCAGTTAAAAATAAGTACTGCATATATAATTACTGG<br>TTTTATATACAGCATAAAAATTACGCCGCTGCGTTTTCCCTGTCAACCCTGT<br>GGATTTTCATTTTTGTGAAAACGATCAAAAAACAACTACTCACAATTCGACA<br>GTCCCGCCAGATACCGCAAAACCGGCCAGACGTTACCGTTTTCCGGGACCA<br>TGATATGAGCCCCGTTGGGGAGGGTATGGAGTTGCTGAAAATGACGGTCAG<br>ATTGAGTTCACCGTTTTATTGTTACAGGAGGCCAGGGCTTGTCTGCTACCGG<br>TCCGGACGAGAGGGATACCGGGAATTTGAATCCGGTTAACTGAGCCGGACA<br>TACGGTAAATAAGGAATGCAGGCAGAACGGGAGTCACTGCAGGATGAGACC<br>GGCACTACAGCAGAAAAACAGGTGATTCTGGTACGGCTCAGCCACTAAACA<br>CAGTTGGAAACTGATGATAAGATAGTCAGCGGTTATATAACTCACTGAGTAA<br>AAAGATATAAAGGTAAGGAGACACTCATGAGTCGTTCCGGTAGCACACTGT<br>ATCTTATCGCCCTGCTGACAGCGGCAACGGTCCTGACAGCCTGCACGCCAA<br>AGGGCAGTATGGAACAACATACCCGGCATTACGTTTATGCATCAGATGACG<br>GTTTTGATCCTAACTTTTCCACCCAGAAGGCCGACACAACA**cgaatgatggtgctttt<br>tttcggcagttctgggatatgggagctaaagataaagcgacaggaaaatcatggagtgatgtgcaacaacgcc<br>ttcagcagtttcacagccaggaatttttaaactcactccggggcacaactcaatttgcgggtaccgattaccgca<br>gcaaagaccttaccccaaaaaaatccaggctgctggctgacaccatttcagcggtttatctcgatggttatgaga<br>gctgacagtgacggggaacgttgcgggggaataaagatattgggggtaggctgagccccagaatatggtac<br>ctccctgcccctccaatattcggtgatggccggacaaggagtccaggagagggggattagtggtggatctgc<br>gccaggtcgtcttcagtctttatgacgctattacggtcaatgccgttctgcagcatggtacgggtaattttcagagt<br>ggcttggagctcactttctgaacgacttattagcgtgtgctccggtgatttgcacatgcgcaaatctttcagccca<br>aaggaggctaatcatcacggcgattacgttcaaatcccccgcgccctcttgctgccaattcagcattgagtacc<br>acgtaacgctcccagcgttcacgcaactgtgcgagctttagcggtaattctttggtgacaaaggcttcctgtattt<br>caaagtcatcaagatgcccttgcagatatgcatgtacgccttccaacacatcagtcagagtgatgttgttactttgt<br>tgcctttcaacaagcggcccctgccagctatgccagaaggcctgatggcgcagacagctttcgtcattcgcga<br>gcaaccacaaatccataaagattcctcgtgcaactttgaacggctatttgtcaacgacggatga	

Supplementary Table 4. Sequence Analysis of the *ΔdapA* deletion

DNA Sequence	Comments
gtaggctgctgcggttatctaaatcgccaacctgcagtttatagtcaccggatgccagacctggatcgc<br>tcgcgcccagttcgtgccattcgctgtcagacagcggcttataagttacggccatgttgccctgcgaac<br>gggtgctgtcggtcactttcatgcccactttttccagcgcagctggcagacgttgccaaaccacattga<br>acggcccgcgtacgaccagcattggtaaaccggtgtcatcagctgcactttgtacgtccatagtggtg<br>gaggcacggttttgcgcagcgttcgcggcgtcagtggcagatttatccagaccggcggaaataacgtt<br>catcatctccgtgctgtaacgctgcatggaagccgcgtctgcaaccggtttgcccgcctgttccaggtt<br>cagcagtttaaccgtaaccgcctgctgataaccctgcggcttaacagagatttgataacgaccacgata<br>ctgctcgtcttcgtccagacggttccattgtacccaatcggtggtcagtgtctgaccagcatcatcacgtt<br>gggtgatggtgtagtttttcgcctgcagcacgctaaccacctgcggccacagagtattgccacgacca<br>ttttccaccagcaatgaagcggtatcgcccgtgaactgggtacgcgcgccagaaaccagtgccagcg<br>gctgggctggtggacgaatgtccagcgccttaccgacggcaccactaccgttggtcaccgggattgc<br>ataatcgccggaggtcaccggcaagatcattccagccggggcatgaagctccgcaagcggtgccgc<br>ttccaggtaggcttcatcaccactgacctgacgcttatagcgtgagtcagaactacaggcagcgagta<br>ataaaacaagcgaaacacccgcaacctttgccaggcgcgacttttgaacagagtaagccatcaaatct<br>ccctaaactttacagcaaaccggcatgcttaagcgccgctctga**CCGTCTCACGACCACTG<br>TCGGTGATTGGTGTCATTGGCAGATTACACGTCTTGAGCGATTGTGTA<br>GGCTGGAGCTGCTTCGAAGTTCCTATACTTTCTAGAGAATAGGAACTT<br>CGGAATAGGAACTAAGGAGGATATTCATATGGACCATGGCTAATTCCC<br>ATGTCAGATCGGAGTAACAATCGCGACAATACTTCCCGTGAACATGGG**<br>ccatcctctgtgcaaacaagtgtctcaatggtacgtttggtatggcattaaaagcaagcagacagaacc<br>gttctgattgttgtatgcatgtttttttatgctttccttaagaacaactcaccccttgaaggaataaccagttt<br>gacactgtcatcgcaacattatctggtgatcactgcgttgggtgccgatcgccctggaatagtgaacac<br>catcacccgtcatgtcagtagttgcggctgtaatattgaagacagtcgcctggcgatgctgggagaag<br>agttcacgtttatcatgctgctttccggttcatggaatgccattactctgattgaatcaacgttaccgttgaa<br>aggtgccgagctggatcttttaatcgtgatgaagcgcacgacggcgcgtccgcgtccgccaatgcca<br>gcatctgtctgggttcaggtcgatgtggcagactccccgcatttaattgaacgcttcacagcacttttcga<br>cacgcatcatatgaacattgcggagctggtgtcgcgcacgcaacctgctgaaaatgaaagggctgcg<br>cagttgcatattcagataactgcccacagccccgcatctgcggacgcagcaaatattgagcaagcgtt<br>caaagccctatgtacagaactcaatgcacaaggcagtattaacgtcgtcaattattcccaacatgatga<br>acaggatggagttaagtaatgaatccactgaaagccggtgatatcgcaccgaaatttagcttgccggat<br>caagacggagaacaagttaatttgaccgacttccagggacagcgtgttctggtttatttctacccgaaa<br>gccatgacccccggctgtaccgtacaggcctgcggcttacgcgataacatggatgagttgaaaaaag<br>cgggcgttgatgtgctgggtatcagcaccgataaacccgaaaaactctcccgttttgcggaaaaagag<br>ctgcttaactttacgctcctgtctgatgagg	The uppercase, bolded blue letters represent the engineered region of the EcN genome, including the EcN sequence homology used for homologous recombination into the EcN genome to create the *dapA* deletion<br><br>Lowercase black letters include EcN sequence upstream and downstream from the intended genomic insertion location<br><br>The entire listed sequence here is confirmed by WGS

Supplementary Table 5. Sequence Analysis of the *ΔΦ* deletion

DNA Sequence	Comments
cgaagatggtcagtgattctttttgccaggtgaacttctctttttcagaaacatgcgtccttccttacgctgcagttac<br>ggtaactttgcagaccgcaacgaaattaccgtcgctggtcataacaataacgtcagcggtgcctgccgccacgc<br>cggtgacggtgatcgcattaccgctaacggtgaccgttgcttttgccccgtctgaggttgccacacggaacgag<br>gtatctgaggcactggctgggttaaccgtcacattgagcgttgtggttgcgccgacggccacgcttgccgtggct<br>ttatcgagcgtaacgcctgtcacggggatattcggggtcccgctttcttctgccagttccggcttgccggtattggt<br>aattttcgctgtacgggttatgacctcttttgccggaatggctttacccaggctgctgcaccagccgcggaaaacg<br>tcgacggtaccgttcgggtatttgattttgtaatagcgtactgagccatcaataaaccatgcgacaaggtctttttgc<br>ccttcttcgcccggcttccaggcgagggtgaacgaggtatcgccagcagattttgccccctgggccgtcgcgtt<br>ccagtcggcatcctcgtcgtcgaggtaagtgtcgtcatacgattcggcggtcatttcgcccggcgtcagctcttta<br>attttcgccaggcggttccagtcgatatccgagagtgggttagcgaaagcgttgcccgttccggtgtaaagccag<br>agggtggtaccggcacctttcacaggggccagcgggtttggagtaggcataagtacctcttaaattgaataggtg<br>attaagtacgtgaaatcgactgaaccccaggtggccatttcatcatcccgctgatagtcataaccctgcggggtg<br>aacgtctcgaccagttcggtcagacctgggatgaaggccattgccggatacactttctcttccatccaggaatcaa<br>gcgcgctgtcggggc**TGGAGGCTTTAAGAAATACCTCGATGTGAACAACCGCCTGC**<br>**CACGAATCTTCGTCAAGCGATTACACGTCTTGAGCGATTGTGTAGGCTGGAG**<br>**CTGCTTCGAAGTTCCTATACTTTCTAGAGAATAGGAACTTCGGAATAGGAACT**<br>**AAGGAGGATATTCATATGGACCATGGCTAATTCCCATGTCAGATCCAGTGAT**<br>**TCTGAATAGGCGATAAGTCCGGTATAACCGGGGATAAT**ctcaccattatcagcttcaaat<br>tcaggaattgtgccggtggtgatggtgtattgaggctggccatcttccttcgcgaaggctgccaggtcttcaatct<br>gcttagctgtaagaactactgtcatgctcattcctcagttgtaaaaaagccccgcgagtgcgaggcgatttgattg<br>aattctcggctcttatctcagcgcagccccttactgcgtgccggttgctcggtgatgagcatcagcgatgagacat<br>taaagccgaccgaaggccagcggcgttcctcatgttgccgacagagccatatcgacaagaggacgaaaacta<br>gcagcatgaatcgcctattggttattcgacagtcgcactgattcgtaaatccgctcacacgtcattcctgcccggta<br>gctttcgtcagatcgtccagcataatatcgagctgcttctgcaaggcttccgagcatgtcggcaagcattgctgcg<br>ttggctccggctgttttgcttctgacggaagtggcgagatctgcggtgtgctttgcggcgtccatgtgggtagcga<br>gttttgttgcttcggcgcgcagctgcttaacagtggtagccaggccagcagaagtaacggcagcgctcgctgctt<br>gagcttgagcatctttaacggcctcatcccgggcgattgttcgcccttgttcaatcatacgagctgcggtctgtgca<br>ttcgcttcctgtgaagattccgcgctatcccggtcagcccactttattttccagctgcggttcgtccactcactgcca<br>gcgagaaacgaacctaccaacgcaacaatcacaatgatgcagatgccacccggcttcactggtctatcccccca<br>gcacgtcagtgcgctttcctggtcccgccgttctacctgcccataacatccatccttctggcccttggtcaggcgg<br>caatcgcggccaccgtctttaatccaccagcggatagcttcacaggctcctttcgtatcgccagcattaa	The uppercase, bolded blue letters represent the engineered region of the EcN genome, including the EcN sequence homology used for homologous recombination into the EcN genome to create the *Φ* deletion<br><br>Lowercase black letters include EcN sequence upstream and downstream from the intended genomic insertion location.<br><br>Entire listed sequence here is confirmed by WGS

Supplementary Table 6. Sequence Analysis of the *ΔthyA* deletion

DNA Sequence	Comments
Atgctccttcagacgaaatccaatctggtcggactcttttaccggttcgcgatcccagataccattactgtt<br>cgcatcccactgcacaatgatacagtcaccctgtccgacaatttccagcccttcgcccgtacagctgcca<br>tggcaataacccgccctctggagatgcttcgcgacggtaaatacccgcagccagatttcatcttccagc<br>gccagcttacgggtgttcgttaaactttcacgctgtaacgcaggcagaaagcgtgcagcccccagcaa<br>caatacgctactgatcgccatagcaatcaacacttccagcagagaaaaaccttgctcttttacaggcatcc<br>ttctgtttctccttgctgacaaagccggagtcttccccacggcgaaaccaccagccaccactcgcccgtt<br>gagtttttgaagcgaatatgcccggcccatgcggtattgcgcaggccaaagaaagcaagcgaaggtgt<br>caggtcgctcatttcgacttcgggccagcgcggcacaaagaccaatggtgaactgccatgacaggtatt<br>cgcccccagcaacggaactcacaaggcaccataacgtcccctccctgataagactgatactgtggtcgc<br>ggttatgccagttggcatcttcacgtaaatagagcaaatagtcccgcgcctggctggcggtttgccaaag<br>ccgttgcgactgctgccagtattgccagccatagagcccacttgcgcttagcatgaccagaatcagcat<br>cgcgaccagcgtttcaatcagcgtataaccacgttgtgttttcatgccggcagtatggagcgaggagaa<br>aaaaagacgagggccagtttctatttcttcggcgcatcttccggactatttacgccgttgcaggacgttgc<br>aaaatttcgggaaggtgtctcgaagaatttaacggagggcaaaaaaaaccgacgcacactggcgtcg<br>gctctggcaggatgtttcgtaattagata**GCCACCGGCGCTTTAATGCCCGGATGTGG**<br>**ATCGTATCCTTCATTACACGTCTTGAGCGATTGTGTAGGCTGGAGCTGC**<br>**TTCGAAGTTCCTATACTTTCTAGAGAATAGGAACTTCGGAATAGGAACT**<br>**AAGGAGGATATTCATATGGACCATGGCTAATTCCCATGTCAGCTTCGTC**<br>**GAGCACTTTTTGCATCAGTTCTAAATACTGTTTC**atggttcctcaggaaacgtgtt<br>gctgtgggctgcgacgatatgcccagaccatcatgatcacacccgcgacaatcatcgggatggaaaga<br>atttgccccatgctgatgtactgcacccaggcaccagtaaactgcgcgtcgggctggcggaaaaactc<br>aacaatgatgcgaaacgcgccgtaaccaatcaggaacaagcctgagacagctcccattgggcgcggt<br>ttacgaatatacaggttgaggataataaacagcaccacaccttccagcagcagctcgtaaagctgtgatg<br>ggtggcgcggcagcacaccgtaagtgtcgaaaatggattgccactgcgggttggtttgcagcagcaaa<br>atatcttctgtacgggagccagggaacagcatggcaaacgggaagttcgggtcaacgcggccccaca<br>attcaccgttaataaagttgcccaggcgcccagcaccaagaccaaacggaatgagtggggcaataaaa<br>tcagagacctggaagaaggaacgtttagtacggcgggcgaagataatcatcaccacgataacgccaat<br>caggcctccgtggaaggacatgccgccgtcccagacacggaaaagatacagcggatcggccataaa<br>ctgcgggaaattgtagaacagaacataaccaatacgtccaccgaggaagacgccgaggaagcccgc<br>atagagtaagttttcaacttcatttttggtccagccactgcccggacgattcgcccgtcgtgttgccagcca<br>cattgcaaaaatgaaacccaccagatacatcaggccgtaccagtgaagcgccacgggtcctattgaga<br>aaatgaccggatcaaactccggaaaatgcagatagctactggtcatctgtcaccacaagttcttgttatttc<br>gctgaaagagaacagcgattgaaatgcgcgccgcaggtttcaggcgctccaaaggtgcgaataatag<br>cacaaggggacctggctggttgc	The uppercase, bolded blue letters represent the engineered region of the EcN genome, including the EcN sequence homology used for homologous recombination into the EcN genome to create the *thyA* deletion<br><br>Lowercase black letters include EcN sequence upstream and downstream from the intended genomic insertion location<br><br>The entire listed sequence here is confirmed by WGS

Supplementary Table 7. Primers for SYNB1891 modified genomic regions

Modified region	Insertion/ Deletion	Primer for Insertion/ Deletion
Exo/cea	P_{fnrS}-*dacA*	CAGACCTTCAGCCAGAGCAG
		CCCAGAAGGCCGACACAACA
ΔΦ	deletion	TGGAGGCTTTAAGAAATACCTCGATGTGAACAACCGCCTGCCACGAATCTTCGTCAAGCGATTACACGTCTTGAGCGAT
		GATAATGGTGAGATTATCCCCGGTTATACCGGACTTATCGCCTATTCAGAATCACTGGATCTGACATGGGAATTAGCCA
ΔdapA	deletion	CGTCTCACGACCACTGTCGGTGATTGGTGTCATTGGCAGATTACACGTCTTGAGCGAT
		CCATGTTCACGGGAAGTATTGTCGCGATTGTTACTCCGATCTGACATGGGAATTAGCCA
ΔthyA	deletion	GGTTCACAGGTTGGATCCTGTCACGCTATAGCTGGCATCCATTACACGTCTTGAGCGAT
		AGAGCAAGGTTTTTCTCTGCTGGAAGTGTTGATTGCCTGACATGGGAATTAGCCA
