# Supplementary material for: Immunotherapy with engineered bacteria by targeting the STING pathway for anti-tumor immunity
Source: Nat Commun. 2020 Jun 1;11:2739. doi: 10.1038/s41467-020-16602-0 (PMC7264239; doi:10.1038/s41467-020-16602-0)
Supplement: Supplementary file 3 — Reporting Summary [file 41467_2020_16602_MOESM3_ESM.pdf]

nature research

Corresponding author(s): Anna Sokolovska, Daniel S. Leventhal and Jose M. Lora

Last updated by author(s): May 1, 2020

Reporting Summary

Nature Research wishes to improve the reproducibility of the work that we publish. This form provides structure for consistency and transparency in reporting. For further information on Nature Research policies, see Authors & Referees and the Editorial Policy Checklist .

Statistics

For all statistical analyses, confirm that the following items are present in the figure legend, table legend, main text, or Methods section.

n/a	Confirmed	
☐	☒	The exact sample size (*n*) for each experimental group/condition, given as a discrete number and unit of measurement
☐	☒	A statement on whether measurements were taken from distinct samples or whether the same sample was measured repeatedly
☐	☒	The statistical test(s) used AND whether they are one- or two-sided<br>*Only common tests should be described solely by name; describe more complex techniques in the Methods section.*
☐	☒	A description of all covariates tested
☐	☒	A description of any assumptions or corrections, such as tests of normality and adjustment for multiple comparisons
☐	☒	A full description of the statistical parameters including central tendency (e.g. means) or other basic estimates (e.g. regression coefficient) AND variation (e.g. standard deviation) or associated estimates of uncertainty (e.g. confidence intervals)
☐	☒	For null hypothesis testing, the test statistic (e.g. *F*, *t*, *r*) with confidence intervals, effect sizes, degrees of freedom and *P* value noted<br>*Give P values as exact values whenever suitable.*
☒	☐	For Bayesian analysis, information on the choice of priors and Markov chain Monte Carlo settings
☒	☐	For hierarchical and complex designs, identification of the appropriate level for tests and full reporting of outcomes
☒	☐	Estimates of effect sizes (e.g. Cohen's *d*, Pearson's *r*), indicating how they were calculated

Our web collection on statistics for biologists contains articles on many of the points above.

Software and code

Policy information about availability of computer code

Data collection	Flow Cytometry: Flow cytometry data was collected on either the Cytoflex LX using the CytExpert Acquistion software (Beckman Coulter) or using the MACS Quant using the MACSQuantify software (Miltenyi Biotec). Multiplexed Cytokine ELISAs: Cytokine bead panels were analyzed using either the MAGPIX analyzer using the MAGPIX analyzer software (Luminex) or the Cytoflex LX using the CytExpert Acquistion software (Beckman Coulter). LC-MS/MS: Mass spectrometry data was collected on the Vanquish UHPLC/TSQ Altis LC-MS/MS system with peaks anlayzed using the Xcalibur Quan Browser (Thermo Fisher Scientific). Miscropscopy: Microscopy was performed using the EVOS™ FL Auto 2 Imaging System and its associated software package (Invitrogen). In Vivo Data Capture: In Vivo measurements and clinical observations were collected using Studylog (Studylog, Pacifica, CA).
Data analysis	Flow Cytometry: Flow cytometry analysis was performed using Flowjo v10 (Becton, Dickinson & Company) or MACSQuantify software (Miltenyi Biotec). Microscopy: Post-acquisition adjustments and quantifications were made using ImageJ v1.8.0 (National Institutes of Health). Statistical Analysis and Graph Generation: Prism7.0c software (GraphPad Software) was used for all statistical analysis.

For manuscripts utilizing custom algorithms or software that are central to the research but not yet described in published literature, software must be made available to editors/reviewers. We strongly encourage code deposition in a community repository (e.g. GitHub). See the Nature Research guidelines for submitting code & software for further information.

Data

Policy information about availability of data

All manuscripts must include a data availability statement. This statement should provide the following information, where applicable:

- Accession codes, unique identifiers, or web links for publicly available datasets
- A list of figures that have associated raw data
- A description of any restrictions on data availability

All data generated or analyzed during this study (including the genome map and manipulated genome sequence of SYNB1891) are available in this published article and its Supplementary Information. Additional data underlying the figures and supplementary information are available from the corresponding authors on reasonable request.

Field-specific reporting

Please select the one below that is the best fit for your research. If you are not sure, read the appropriate sections before making your selection.

- [x] Life sciences
- [ ] Behavioural & social sciences
- [ ] Ecological, evolutionary & environmental sciences

For a reference copy of the document with all sections, see nature.com/documents/nr-reporting-summary-flat.pdf

Life sciences study design

All studies must disclose on these points even when the disclosure is negative.

Sample size	Sample size was determined empirically, with variations between samples utilized to determine proper number of samples to reach sufficient statistical power for the analyses performed. To determine the adequate number of samples/replicates per group, we assumed an effect size of at least 30% (with a 10% difference being biologically meaningful), a desired power of 90%, significance level of $p < 0.05$ and utilization of two-sided testing to compare experimental to control groups. This resulted in a need for group sizes of 3-5 replicates/animals per experimental group. For efficacy studies utilizing murine tumor models, numbers were increased to 10 mice per group due to the increased variability in tumor growth and potential for loss of mice prior to study endpoints.
Data exclusions	No data were excluded from the analyses in this study.
Replication	All reported findings were replicated at least once (e.g. n greater than or equal to 2), with data shown being consistent and representative of two or more independent experiments. All attempts at replication were successful.
Randomization	All In Vitro studies utilized homogeneous mixtures of bacterial or mammalian cells which were allocated into various experimental groups and treated equally except for the imposed experimental variable as stated, thus no randomization was required for such experiments. For In Vivo studies, age-matched and co-housed mice were randomized into treatment groups based on tumor volume in order to create experimental groups with approximately equal average tumor volumes at the initiation of treatment.
Blinding	Since the same researchers performing treatments were also responsible for planning and analysis of experiments, they were not blinded to allocation during experiments or outcome assessments.

Reporting for specific materials, systems and methods

We require information from authors about some types of materials, experimental systems and methods used in many studies. Here, indicate whether each material, system or method listed is relevant to your study. If you are not sure if a list item applies to your research, read the appropriate section before selecting a response.

Materials & experimental systems

n/a	Involved in the study	
[ ]	[x]	Antibodies
[ ]	[x]	Eukaryotic cell lines
[x]	[ ]	Palaeontology
[ ]	[x]	Animals and other organisms
[x]	[ ]	Human research participants
[x]	[ ]	Clinical data

Methods

n/a	Involved in the study	
[x]	[ ]	ChIP-seq
[ ]	[x]	Flow cytometry
[x]	[ ]	MRI-based neuroimaging

Antibodies

Antibodies used	Microscopy: primary antibody against GFP (rabbit anti-GFP, Abcam, Cat. # ab6556) and LAMP-1 (rat anti-mouse-LAMP-1 (1D4B), eBioscience, Cat. # 14-1071-82) with secondary antibody donkey anti-rabbit IgG (Alexa488, ThermoFisher Scientific, Cat. # A-21206) and goat anti-rat IgG (Alexa 647, ThermoFisher Scientific, Cat. # A-21247). In Vivo T cell depletion: rat IgG2b isotype

	control (clone: LTF-2, BioXcell, Cat. # BP0090), anti CD4 (clone: GK1.5, BioXcell, Cat. # BP0003-1) or anti CD8α (clone: 2.43, BioXcell, Cat. # BP0061)
Validation	Anti-GFP from Abcam was validate by western blot comparison of null cell lines compared to GFP over expressing cell lines. Anti Lamp-1 from eBioscience was validated in the literature and by the manufacturer via flow cytometry staining of BALB/c thioglycolate-induced peritoneal exudate cells compared to isotype control. Thermofisher Scientific secondary antibodies, anti-rabbit and anti-rat, were highly cross-absorbed and shown to exhibit minimum cross-reactivity to bovine, chicken, goat, guinea pig, hamster, horse, human, mouse, rat, and sheep serum proteins. All BioXcell antibodies were validate for binding specificity to mouse CD4 or CD8α by manufacturer via flow cytometry, ELISA, and/or Western blot. For BioXcell depletion antibodies, the antibody dose level, dose frequency, ability to deplete target cells and specificity for target cells have been evaluated extensively previously in the literature and were confirmed in a pilot assay before full scale experiments were performed.

Eukaryotic cell lines

Policy information about cell lines

Cell line source(s)	All cells lines used in this study were acquired from ATCC (B16.F10 melanoma (ATCC, CRL-6475), EL4 T-cell lymphoma (ATCC, TIB-39), A20 B-cell lymphoma (ATCC, TIB-208), 4T1 mammary carcinoma (ATCC, CRL-2539) and CT26 colon carcinoma (ATCC, CRL-2638), Murine RAW264.7 macrophage cell line (ATCC, TIB-71)) or InVivoGen (THP-1 cells lines containing the following TMEM173 (STING) alleles: H232 (InVivoGen, thpd-h232), HAQ – THP1 endogenous allele (InVivoGen, thpd-nfis) and R232 (InVivoGen, thpd-r232)).
Authentication	Cell line authentication and mouse pathogen screening was carried out via IMPACT testing through IDEXX BioResearch.
Mycoplasma contamination	Cell line authentication and mouse pathogen screening was carried out via IMPACT testing through IDEXX BioResearch. All cell lines tested negative for contamination with mycoplasma.
Commonly misidentified lines (See ICLAC register)	No commonly misidentified cell lines were used in this study.

Animals and other organisms

Policy information about studies involving animals; ARRIVE guidelines recommended for reporting animal research

Laboratory animals	Specific pathogen free C57BL/6, BALB/c, STING-/- (C57BL/6J-Tmem173gt/J) and TLR4-/- (B6.B10ScN-Tlr4lps-del/JthJ) female mice were purchased from Jackson Laboratory and were utilized or placed on study at 7-to-9 weeks-of-age.
Wild animals	No wild animals were used in this study.
Field-collected samples	No field collected samples were used in this study.
Ethics oversight	Animal housing and all procedures related to in vivo experiments were reviewed and approved by Mispro's Institutional Animal Care and Use Committee (Mispro Biotech Services, 400 Technology Square, Cambridge, MA, 02139) in accordance with the Animal Welfare Act.

Note that full information on the approval of the study protocol must also be provided in the manuscript.

Flow Cytometry

Plots

Confirm that:

- [x] The axis labels state the marker and fluorochrome used (e.g. CD4-FITC).
- [x] The axis scales are clearly visible. Include numbers along axes only for bottom left plot of group (a 'group' is an analysis of identical markers).
- [x] All plots are contour plots with outliers or pseudocolor plots.
- [x] A numerical value for number of cells or percentage (with statistics) is provided.

Methodology

Sample preparation	Tumors were harvested from mice and placed into single cell suspension by mechanical disruption using a 40uM cell strainer. Tumor cells were pelleted by centrifugation and resuspended in PBS for analysis.
Instrument	MACS Quant (Miltenyi Biotec).
Software	MACSQuantify software (Miltenyi Biotec).
Cell population abundance	No sorting was conducted and who tumor single cell suspensions were analyzed.

Gating strategy	SSC and FSC thresholds were reduced to allow for visualization of small bacterial cells within tumor homogenates. RFP+ bacterial cells were gated on by plotting SSC vs RFP (mCherry channel). GFP+ among RFP+ bacteria were then quantified by plotting RFP (mCherry channel) vs GFP (FITC channel).

☒ Tick this box to confirm that a figure exemplifying the gating strategy is provided in the Supplementary Information.
